# Potential Anticancer Effects of Polyphenols from Chestnut Shell Extracts: Modulation of Cell Growth, and Cytokinomic and Metabolomic Profiles

**DOI:** 10.3390/molecules21101411

**Published:** 2016-10-21

**Authors:** Angela Sorice, Francesco Siano, Francesca Capone, Eliana Guerriero, Gianluca Picariello, Alfredo Budillon, Gennaro Ciliberto, Marina Paolucci, Susan Costantini, Maria Grazia Volpe

**Affiliations:** 1 CROM, Istituto Nazionale Tumori “Fondazione G. Pascale”—IRCCS, Napoli 80131, Italy; a.sorice@istitutotumori.na.it (A.S.); f.capone@istitutotumori.na.it (F.C.); e.guerriero@istitutotumori.na.it (E.G.); a.budillon@istitutotumori.na.it (A.B.); 2 Consiglio Nazionale delle Ricerche, Istituto di Scienze dell’Alimentazione, Via Roma 64, Avellino 83100, Italy; francesco.siano@isa.cnr.it (F.S.);picariello@isa.cnr.it (G.P.); 3 Direttore Scientifico, IRCCS Istituto Nazionale Tumori “Fondazione G. Pascale”, Napoli 80131, Italy; g.ciliberto@istitutotumori.na.it; 4 Dipartimento di Scienze e Tecnologie, Università degli Studi del Sannio, Via Port’Arsa 11, Benevento 82100, Italy; paolucci@unisannio.it

**Keywords:** chestnut shells extract, polyphenols, cancer

## Abstract

In this study, a hydroalcoholic chestnut shell extract was characterized and tested on six different human cell lines. Gallic, ellagic, and syringic acids were the most abundant non-condensed compounds in the chestnut extract, as determined by high performance liquid chromatography (HPLC). Tannins were mainly represented by condensed monomeric units of epigallocatechin and catechin/epicatechin. After 48 h of treatment, only the human hepatoblastoma HepG2 cells reached an inhibition corresponding to IC_50_ with an increase of apoptosis and mitochondrial depolarization. The cytokinome evaluation before and after treatment revealed that the vascular endothelial growth factor (VEGF) and the tumor necrosis factor (TNF)-α decreased after the treatment, suggesting a potential anti-angiogenic and anti-inflammatory effect of this extract. Moreover, the metabolome evaluation by ^1^H-NMR evidenced that the polyphenols extracted from chestnut shell (PECS) treatment affected the levels of some amino acids and other metabolites. Overall, these data highlight the effects of biomolecules on cell proliferation, apoptosis, cell cycle and mitochondrial depolarization, and on cytokinomics and metabolomics profiles.

## 1. Introduction

Plants, including most food and feed plants, produce a broad range of bioactive chemical compounds via their so-called secondary metabolism. Polyphenols constitute one of the most numerous and ubiquitous groups of such compounds and are an integral part of both human and animal diets [1,2]. Nowadays, polyphenols have received considerable interest due to their therapeutic effects. Indeed, evidence exists pointing at the biological effects of polyphenols on the prevention of various pathologies, including cardiovascular diseases, neurodegenerative disease, and cancer [3]. Several epidemiological studies have shown that polyphenols inhibit or attenuate the initiation, progression, and spread of different types of cancers in experiments in vitro and in vivo [4]. Their anticancer activities are polyhedral and affect regulation of growth factor-receptor interactions and cell signaling cascades concerning the genes’ expression involved in cell cycle arrest, cell survival, angiogenesis, and apoptosis [4]. Studies aimed to characterize polyphenols, and their metabolic pathways, showed important results in cancer treatment and prevention both in tumor cell lines in vitro, in murine models in vivo, and in human trials [5].

Polyphenols are present in almost all classes of food and agro-industrial residues. The by-products of fruits, vegetables, oilseeds, nuts, and cereals have been found to contain high amounts of polyphenols [6].

With an average production of 50,000 tons/year, Italy is the main chestnut (*Castanea sativa*) producing country in Europe [7]. Within Italy, Italian Institute of Statistics (ISTAT) data show that the Campania region is the area with the highest chestnut production and export. Chestnuts are rarely consumed raw. During chestnut processing, a large amount of waste is produced, mainly represented by the burr and the shell. The shell is generated in the peeling process and, nowadays, it is used as fuel in the factory, especially in Spain [8]. Several studies have shown the presence in the chestnut waste of bioactive molecules with positive effects on health and human welfare [9,10,11,12]. The analysis of chestnut leaves, flowers, skins, and fruits has highlighted the presence of bioactive molecules of a phenolic nature, characterized by good antioxidant activity [10,13,14]. The exploitation of these wastes would reduce their environmental impact and an economic benefit could also be obtained. Indeed, antioxidants and other bioactive components, such as polyphenols present in shells, wood, and leaves are used in animal feeds [15]. Other possible applications of the phenolic compounds include their use as ingredients in functional foods, as nutraceuticals, and antimicrobial agents [16,17,18].

Advantages of polyphenols as anticancer agents are their high accessibility, low toxicity, and specificity of the biological response [3]. A combination of cytoprotective effects toward normal cells and cytotoxic effects toward cancerous cells thus represents the main advantage of polyphenols as anticarcinogenic agents [19]. For example, Brizi et al. [20] have highlighted the neuroprotective effect of *Castanea sativa* Mill. polyphenols on human neuroblastoma cells evidencing a role on neurodegenerative disease. Since little information is reported about phenolic chestnut shell extracts [21,22,23], in this preliminary study a hydroalcoholic chestnut shell extract was characterized and tested on the human normal keratinocyte HaCaT cell line, and on five different human tumor cell lines (the human malignant melanoma A375 cell line, the human lung cancer H460 cell line, the human liver hepatocellular carcinoma HepG2 cell line, the human colorectal adenocarcinoma HT29 cell line, and the human estrogen-receptor-positive breast cancer MCF7 cell line). Cell growth, cell death, cell cycle modifications, and mitochondrial membrane depolarization were analyzed. Moreover, after cell treatment with polyphenols extracted from chestnut shell (PECS), the tumor microenvironment immunomodulation by the cytokines profile was analyzed, and the metabolites were evaluated by NMR.

## 2. Results

### 2.1. PECS Composition

The antioxidant activity (% inhibition) of PECS, and that of gallic acid used as a reference, were 78.5 ± 1.1 and 82.6 ± 0.9, respectively. The concentration of total phenolic compounds in PECS was 590.2 ± 1.7 g/kg of dried extract, while the yield (mg extracted solid/g of chestnut shell) was 13.5%. The polyphenols in the extract were quantified by the external standard method, comparing the peak area of isolated components to that of the corresponding pure standard. 

The reference multistandard mixture consisted of eight pure polyphenols: gallic acid (1); caffeic acid (2); chlorogenic acid (3); syringic acid (4); ferulic acid (5); ellagic acid (6); rutin (7), and quercetin (8). High performance liquid chromatography (HPLC) separations were monitored at 275, 325, and 375 nm in order to selectively detect general phenolics, hydroxycinnamic acid derivatives, and flavonols/ellagic acid, respectively. Therefore, gallic acid was determined in the chromatogram at 275 nm, and syringic acid at 325 nm, whereas ellagic acid, rutin, and quercetin were quantified at 375 nm. The most abundant phenolic compound in chestnut peel extracts was gallic acid, (2.12 ± 0.15 g/Kg of dry weight), followed by ellagic acid (1.05 ± 0.18 g/Kg) and syringic acid (0.50 ± 0.10 g/Kg). Quercetin and rutin had lower concentrations (0.081 ± 0.010 and 0.059 ± 0.007 g/Kg of dry weight). Quantification of syringic acid is approximate, because it is biased by the presence of interfering condensed tannins. 

**Figure 1:**
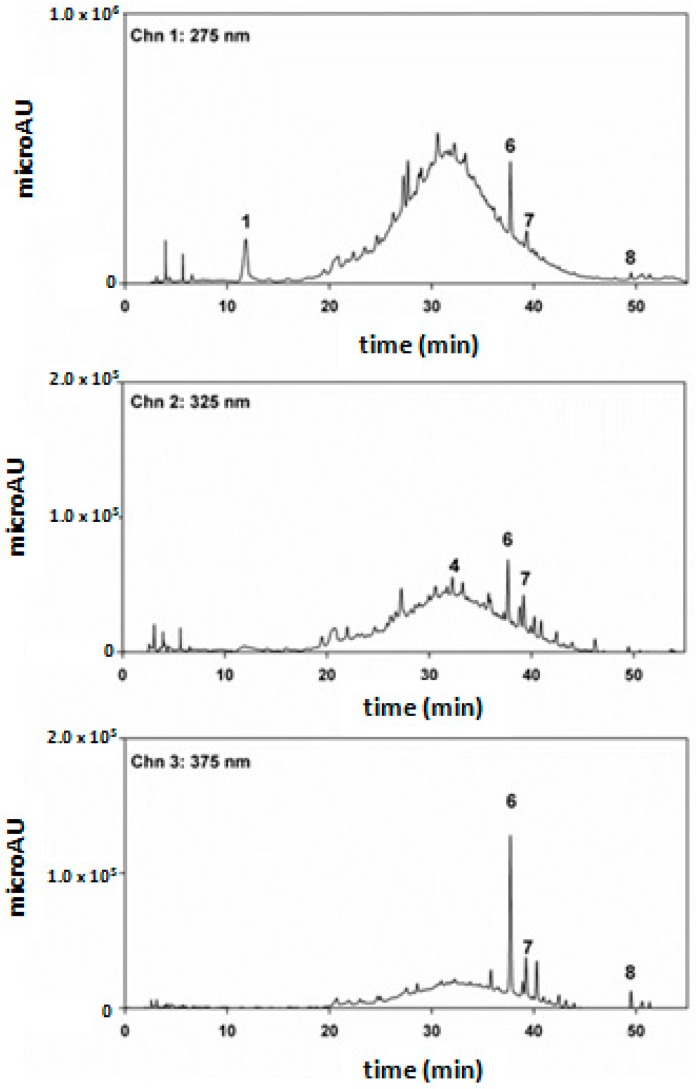
Chromatographic separation (HPLC/DAD) of chestnut shell extract monitoring atthe wavelengths of 275, 325, and 375 nm. The numbers indicate the following molecules: gallic acid (1), syringic acid (4), ellagic acid (6), rutin (7), and quercetin (8).

**Figure 2:**
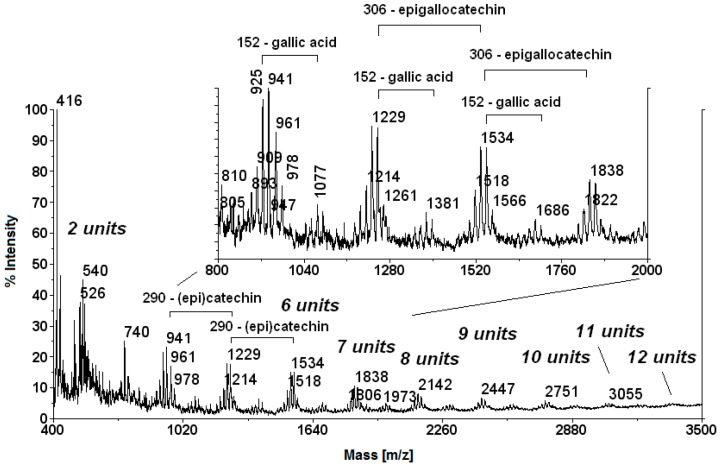
MALDI-TOF MS spectrum of the chestnut peel extract acquired in the linear ion mode. Up to 12 condensed monomeric units were detected. A shift of 1–2 units in the measurement of the MW are within the ordinary experimental error of the linear mode MALDI-TOF MS analysis.

The HPLC chromatograms at 275 nm was dominated by a broad and unresolved peak due to a multitude of condensed oligomers containing catechin/epicatechin, epigallocatechin, and epicatechingallate as monomeric units [24]. Chromatograms at 325 and 375 nm exhibited a progressive disappearance of the broad peaks of condensed tannins and the increase of sharp peaks corresponding to ellagic acid, rutin, and quercetin (Figure 1). The dominance of oligomeric condensed tannins was confirmed by direct matrix assisted laser desorption ionization-time of flight (MALDI-TOF)/mass spectrometry (MS) analysis (Figure 2).

### 2.2. Effect of PECS on Cell Viability

**Figure 3:**
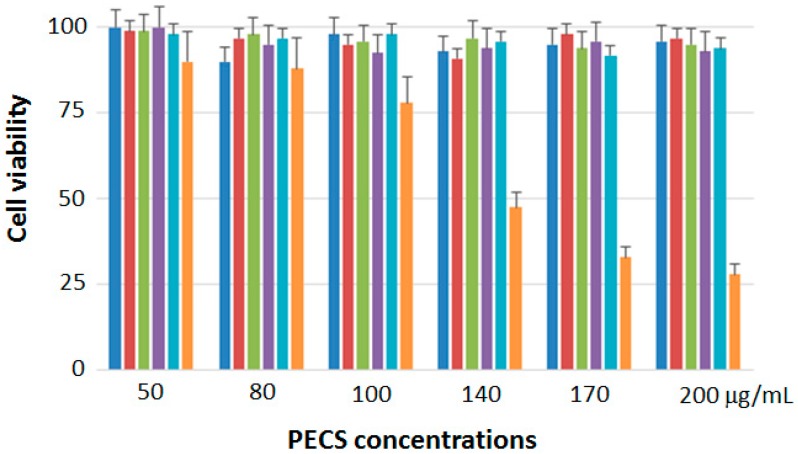
Cell viability related to six cell lines, A375 (in blue), H460 (in red), MCF7 (in green), HT29 (in violet), HaCaT (in cyan), and HepG2 (in orange), after the treatment with the phenolic extract (PECS) for 48 h.

The possible cytotoxic effect of PECS was evaluated on six cell lines by Sulforhodamine B (SRB) assay to identify the concentrations at which the cell growth was inhibited by 50% (IC_50_). After 48 h of treatment, compared to untreated cells, HaCaT, A375, MCF7, HT29, and H460 cells retained a relatively constant viability, whereas only HepG2 cells showed a growth inhibition with an IC_50_ of 137 μg/mL (Figure 3). It is important to report that HepG2 cells are a pure cell line of human liver carcinoma, often used as a model. It was derived from the liver tissue of a fifteen year old Caucasian American male with a well-differentiated hepatocellular carcinoma. These cells are epithelial in morphology and hepatitis B virus surface antigens have not been detected. Based on these considerations and obtained results, the following studies were performed only on the HepG2 cell line with the future aim to test PECS effects on other human liver cancer cell lines that are reported as more undifferentiated and aggressive [25].

### 2.3. Apoptosis Increase in HepG2 Cells

To further investigate the cause of the decrease of cell viability induced by PECS, the cells were labeled with an Annexin V and Dead Cell Assay kit that was used to detect apoptotic cells. As shown in Table 1, after 48 h of treatment with 137 μg/mL of PECS, a late apoptosis increase was observed in HepG2 cell line corresponding to 52.21%, compared to untreated cells at 4.81%. Further studies will regard the validation of these results by the evaluation of the expression of the apoptosis molecules by Western blotting experiments.

### 2.4. Modulation of Cell Cycle in HepG2 Cells

The effects of the polyphenolic extract on the cell cycle was studied by Muse™ Cell Cycle Assay after 48 h of treatment with 137 μg/mL of PECS. A mild elongation in the cells’ percentage at the G0/G1 phase was observed (61.8%), accompanied by a corresponding reduction in the cells’ percentage in S (3.9%) and G2/M phases (33.0%) compared to the control (Table 2). However, these results will be verified also by the evaluation of the expression of cyclin D1 by Western blotting experiments.

### 2.5. Mitochondrial Membrane Depolarization in HepG2 Cells

The human liver hepatocellular carcinoma cell line HepG2 was treated with PECS, and their effects on the depolarization of the mitochondrial membranes is understood as loss of DYm via a Muse MitoPotential Assay Kit. Loss of the mitochondrial inner transmembrane potential is a consistent indicator of mitochondrial dysfunction and cellular health. An increase in the mean percentage of cellular depolarization was observed in the presence of IC_50_ concentrations of PECS, compared to non-treated cells (Table 3). Moreover, a slight increase of dead cells was also observed when the cells were treated with PECS, compared to those left untreated. 

### 2.6. Decrease of VEGF and TNF-α Levels in HepG2 Supernatants

The levels of 27 cytokines in HepG2 supernatants after treatment with PECS were evaluated by multiplex biometric ELISA-based immunoassay. The comparison between treated and untreated cells evidenced that only vascular endothelial growth factor (VEGF) and tumor necrosis factor (TNF)-α decreased after the treatment (Table 4). No other modifications were detected.

### 2.7. Identification of Key Differential Metabolites

^1^H-NMR spectra were obtained on the aqueous phases that were extracted from HepG2 cells before and after PECS treatment. Table 5 shows the proton resonances related to the metabolites identified in HepG2 cells. In detail, the spectral region from 0.5–3 ppm contains signals from leucine, valine, threonine, isoleucine, alanine, lysine, arginine, glutamine, glutamate, glutathione, aspartate, lactate, acetate, and malate. The spectral region from 3–5.5 ppm contains mainly signals form creatine, phosphorylethanolamine, phosphocholine, choline, myo-inositol, α-glucose, β-glucose, glycine, glycerol, glycero-phosphocholine, glycogen, and creatine. The 5.5–8.5 ppm region contains the resonances of uracil, fumarate, histidine, tyrosine, phenylalanine, and formate.

**Figure 4:**
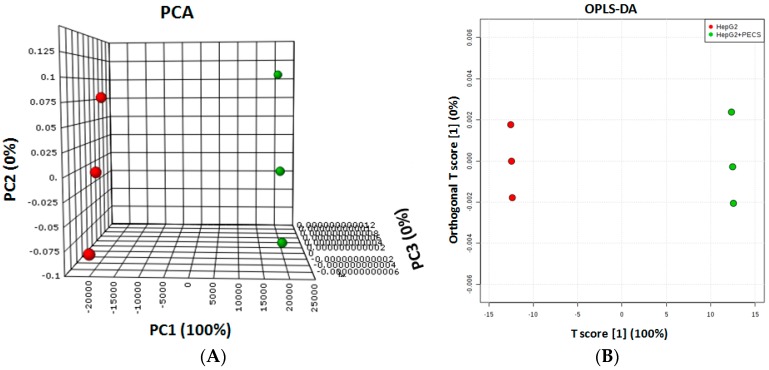
PCA (**A**) and OPLS-DA (**B**) plot obtained for HepG2 cells in triplicate, before (red circles) and after (green circles) PECS treatment.

Principal component analysis (PCA) and orthogonal projections to latent structures discriminant analysis (OPLS-DA) were used to extract the systematic variation in a data matrix, to identify trends and cluster, and to perform a discriminant analysis. PCA and OPLS-DA plots showed that the aqueous extracts from HepG2 cells, before and after PECS treatment, grouped into two different clusters with a clear separation between them (Figure 4A,B).

**Figure 5:**
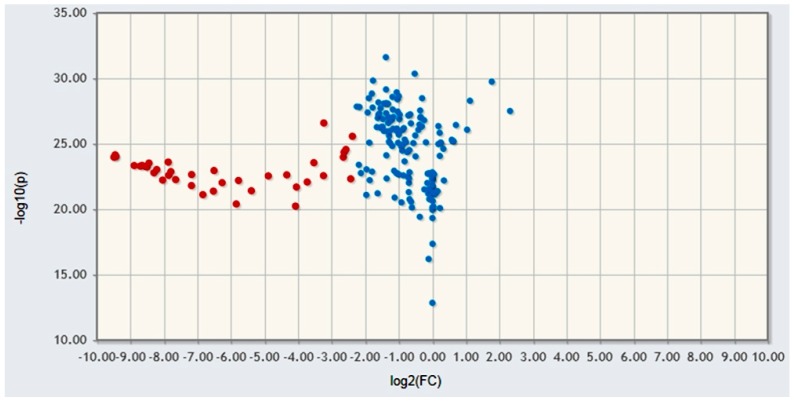
Volcano plot shows the logarithm of fold change (FC) obtained as the ratio from the peak intensities of the all of the protons in the ^1^H spectra evaluated in treated vs. untreated HepG2 cells with respect to the logarithm of *p*-values by *t*-test analysis. Red symbols show the resulting protons that are statistically different between treated and untreated HepG2 cells by using, as threshold values, 2 for fold changes and 0.05 for *p*-values.

The fold change analysis and *t*-test were used to identify major metabolites able to differentiate our treated and untreated samples. As visible from the volcano plot (Figure 5), the metabolite profile of HepG2 cells treated with PECS was clearly discriminated from the control. Ten amino acids were significantly decreased after 48 h of treatment (alanine, arginine, aspartate, glutamine, glutamate, histidine, leucine, lysine, tyrosine, and valine) as well as the following six molecules: alpha and beta glucose, glutathione, lactate, malate, and uracil.

## 3. Discussion

Many studies conducted on *Castanea sativa* by-products reveal a high content of polyphenols [9,11,13,26,27,28,29]. Under our extraction conditions the recovery of the phenolic components was around 590 g/kg of dry extract, a value similar to that obtained by Vázquez et al. [30], and well above what was reported by Noh et al. [31]. It is likely that such discrepancies are due to the methodology employed. The solubility of polyphenols is influenced by the polarity of the solvents used. Solvents such as water, methanol, ethyl acetate, and ethanol-aqueous solutions are all suitable agents for the extraction of polyphenols from *Castanea sativa* by-products and have been employed by different authors [32,33,34,35,36].

Water is a biorenewable non-toxic solvent, normally used for the extraction of antioxidant compounds from chestnut by-products [27]. However, high phenolic polymers and tannins from chestnut shells can be found in water fractions, as occurred in our chestnut shell extracts. Ethanol is considered a good solvent for polyphenol extraction and safe for human consumption [37] even if very polar phenolic acids (benzoic, cinnamic acids) could not be extracted completely, and, hence, mixtures of alcohol–water can be found. 

Working temperature and time length of extraction are crucial factors that should be taken into consideration during the extraction processing. Many phenolic compounds are subjected to degradation or undergo undesirable oxidation at high temperatures and during prolonged extraction periods [38].

In agreement with a previous characterization [24], MALDI-TOF/MS reflects the complexity of the condensed oligomeric proanthocyanidin which contained up to 12 or more monomeric units of randomly-distributed (*epi*)catechin (MW = 290), epigallocatechin (MW = 306), and epigallocatechin gallate (MW = 442). The structure of oligomeric proanthocyanidins could be even more complex as found in chestnut bark [26], due to two possible typologies of interflavan linkages of flavan-3-ol units, namely A- and B-type, which are not distinguished by mass spectrometry. A series of ellagitannins, which have been already characterized in chestnut shells and after biosynthesis, probably escaped both HPLC and MALDI MS-based detection due to suppression effects of dominant oligomeric proanthocyanidins.

Six different human cell lines, HaCaT, A375, H460, HepG2, HT29, and MCF7, have been exposed to PECS, and IC_50_ was reached only in the HepG2 cell line. The polyphenolic treatment showed to have cytotoxic effects only on the liver cancerous cells line, HepG2 cells, in accordance with previous papers reporting that procyanidins from *Castanea mollissima Bl.* shells induced a significant decrease in the inhibition rate of cell proliferation in HepG2 cells [17]. Since PECS induced a cytotoxic effect only on HepG2 cells, its ability to induce death, mitochondrial membrane depolarization, and modification of cell cycle phases, to modulate tumor microenvironment by cytokines profiling, and to modify the metabolite expression by the NMR approach, was evaluated only on this cell line. In particular, our results evidenced a late apoptosis increase after PECS treatment on HepG2 cells, in agreement with Lee et al. [18], which showed a strongly retarded cell proliferation and apoptosis induction by chestnut powder in the AGS gastric cancer cell line. Our data did not show an early apoptosis but did show, directly, a late apoptosis signal that could represent an “apoptosis-necrosis continuum” [39]. In fact, many indications show that these processes represent morphologic expressions of a shared biochemical network [39].

Moreover, PECS showed a modulatory action on cell cycle progression in HepG2 cells, in agreement with data in the literature reporting that procyanidins from *Castanea mollissima* induced an arrest of the cell cycle in the G0/G1 phase with a concurrent reduction of cells in the S phase on HepG2 cells [17]. Such behavior could be explained by the activation of a different pathway of cell death that does not see the involvement of mitochondria. Recently, Zhang et al., in two different papers, have highlighted that procyanidins from chestnut (*Castanea mollissima Bl*.) shells caused the loss of mitochondrial membrane potential and stimulated reactive oxidative species (ROS) generation in human hepatoma HepG2 cells [17,18,19]. Our outcome on the mitochondrial membrane potential increase in HepG2 cells after PECS treatment seems to confirm the data already reported in the literature.

Hence, in summary, the coexistence of an “apoptosis-necrosis continuum”, mitochondrial membrane potential increase, and mild G0/G1 phase elongation highlight how the cells respond to the PECS treatment and what cellular mechanisms are modulated and activated from it.

Several studies reported that polyphenolic extracts from different natural sources show anti-inflammatory and anti-angiogenic effects [40,41,42]. In this study the evaluation of the cytokinome profile of human hepatoma HepG2 cells evidenced a modulation of vascular endothelial growth factor (VEGF) and tumor necrosis factor (TNF)-α after PECS treatment. VEGF is a potent angiogenic factor that is up-regulated in many tumors and its contribution to tumor angiogenesis is well defined [43]. However, its high serum levels indicate a poor prognosis for patients with hepatocellular carcinoma (HCC) [44,45]. TNF-α is a cell signaling cytokine involved in inflammation sites and acute phase reaction [46]. Recently, water chestnut extract has been demonstrated to inhibit the inflammatory cytokine secretion such as TNF-α [47]. Therefore, our results suggest an anti-angiogenic and anti-inflammatory role of PECS on HepG2 cells.

On the other hand, the evaluation of the metabolomic profile also suggests a reduction of some amino acids, as well as other molecules, in HepG2 cells treated with PECS. Therefore, our data can highlight a chestnut shell polyphenol-dependent activation of different metabolic processes able to produce energy. In particular, the following metabolic pathways resulted in being modulated from the PECS treatment: aminoacyl-tRNA biosynthesis, nitrogen metabolism, pantothenate and coA biosynthesis, beta-alanine metabolism, arginine and proline metabolism, d-glutamine and d-glutamate metabolism, cyanoamino acid metabolism, alanine, aspartate and glutamate metabolism, valine, leucine and isoleucine biosynthesis, glycolysis or gluconeogenesis, lysine biosynthesis, and propanoate metabolism. Specific amino acid dependency is one of the metabolic abnormalities of cancer cells and can also be regarded as the metabolic basis for their malignant behavior [48], implying that the malignant behaviors of cells could be dependent on, or related to, specific amino acids. For example, leucine and valine play important roles in protein synthesis and serve as major nitrogen donors for alanine and glutamine synthesis. Increasing evidence shows that, especially leucine regulates the mammalian target of rapamycin (mTOR) pathway that is up-regulated in many cancer types and mTOR-targeted cancer therapy has been a part of clinical research [48]. Moreover, glutamine is an important energy source and products of its metabolism include glutamate and glutathione, molecules that play a crucial role in tumor proliferation, invasiveness, and resistance to therapy [49]. High levels of glutamate in HepG2 cells were associated to increased proliferation of this cell line [49]. This is in agreement with our results, showing a decrease of glutamate production after PECS treatment in HepG2 cells and a reduction of HepG2 cells proliferation.

Finally, most tumors are highly dependent on glucose in support of bioenergetic and macromolecular synthesis. It has been known since the 1920s [50] that tumor cells have a higher rate of glucose consumption through a different glycolysis pathway in which there is the conversion of pyruvate to lactate. Thus, glucose consumption is an important step for cancer cells because this deprivation can induce their death or necrosis [51,52]. In this respect the effect of PECS on glucose diminution in human hepatoma HepG2 cells is remarkable. 

In conclusion, all our data show, for the first time, the effects of polyphenols from chestnut shells on cell proliferation, apoptosis, cell cycle, and mitochondrial depolarization, but also point to the capacity of polyphenols to modulate both the cytokinomic and the metabolomic profiles of HepG2 cells.

## 4. Materials and Methods

### 4.1. Extraction and Concentration of Polyphenols from Chestnut Shell

Extraction of polyphenols from chestnut shell was carried out with 70% ethanol for 3 h at room temperature. The polyphenols extracted from chestnut shells (PECS) were recovered by vacuum filtration and the solvent was evaporated in a rotary evaporator (Mod. Hei VAP Value; Heidolph, Schwabach, Germany). The residue was placed in a drier and weighed up to a constant value and the extraction yield was calculated as the percentage weight loss of the starting material.

### 4.2. Antioxidant Activity

The antioxidant activity of the PECS was evaluated as the antiradical activity by using the free radical 2,2-diphenyl-1-picrylhydrazyl (DPPH^•^), according to Barreira et al. [13] One-hundred milligrams (100 mg) of extract were added to 2.4 mL of 0.0004% DPPH^•^ in methanol and the absorbance was measured at 517 nm until the reaction reached a plateau (1 h). The anti-radical activity was expressed as a percentage of inhibition (%*I*) of the sample (*A*s) compared to the initial concentration of DPPH^•^ (*A*c) according to the equation: %*I* = [(*A*c − *A*s)/*A*c] × 100. The analyses were done in triplicate.

### 4.3. Total Phenols Content

Total phenols content in PECS was determined by the Folin-Ciocalteu method [53]. Two and a half five milliliters (2.5 mL) of Folin-Ciocalteu reagent, previously diluted with water (1:10, *v*/*v*), and 2 mL of 75 g/L aqueous solution of sodium carbonate were added to 0.5 mL of an aqueous solution of the extract. The mixture was kept for 5 min at 50 °C and, after cooling, the absorbance at 760 nm was measured. The total phenols content was calculated as gallic acid equivalents from the calibration curve of gallic acid standard solutions (2–40 mg/mL) and expressed as mg gallic acid equivalent (GAE)/mg of extract (on a dry basis). The analyses were done in triplicate.

### 4.4. HPLC Analyses of Polyphenols

Polyphenols in PECS were detected by HPLC-DAD. The method described by Li et al. [54] was used with some modifications. Surveyor HPLC system (Thermo Finnigan, San Diego, CA, USA) equipped with a pump, a degasser, a thermostatic autosampler, and a photodiode array detector (DAD) was used. The separation was carried out in an Ultra Phenyl (150 × 4.6 mm; particle size: 5 μm; pore size: 100 Å) Resteck column. Samples and standards were filtered using a 0.45 mm Agilent microfilter (Agilent Technologies, Milano, Italy). The binary mobile phase consisted of acetonitrile (solvent A) and water containing 2% acetic acid (solvent B). The system was run with a gradient program: 95% B for 10 min, 95% to 60% B in 35 min, 60% B to 45% B in 10 min, and 45% B to 95% B in 10 min. The flow rate was kept constant at 1.0 mL/min for a total run time of 65 min. The sample injection volume was 10 μL. Peaks of interest were monitored at 275, 325, and 375 nm. Polyphenols extracted were identified by comparing retention times of the detected peaks and UV-VIS spectra with those of pure commercial standards and with literature data.

### 4.5. MALDI-TOF/MS

PECS were analyzed by matrix assisted laser desorption ionization-time of flight (MALDI-TOF) MS analysis using a Voyager DE-Pro (PerSeptiveBiosystems, Framingham, MA, USA). Prior to MS analysis, aliquots of extracts were purified by Zip-Tip (Millipore, Bedford, MA, USA) C18 reversed-phase pre-packed microcolumns, washed with 0.1% (*v*/*v*) trifluoroacetic acid, and eluted with 50% acetonitrile (*v*/*v*) containing 0.1% TFA. Spectra were acquired in positive linear and reflector ion modes using 2,5-dihydrobenzoic acid (DHB) as the matrix (10 mg/mL in 50% acetonitrile, *v*/*v*). Typically, the *m*/*z* 400–4000 range was explored. The mass ranges was externally calibrated with a mixture of standard peptides (Sigma Aldrich, St. Louis, MO, USA). Spectra were elaborated using the Data Explorer 4.0 software (Applied Biosystems Inc., Lincoln Centre Drive Foster City, CA, USA) furnished with the spectrometer. 

### 4.6. Cell Culture and Treatment

The six cell lines, A375, H460, HT29, MCF7, HepG2, and HaCaT (Lonza, Verviers, Belgium), were kept in culture and expanded at 37 °C in a humidified atmosphere of 5% CO_2_ in DMEM (Dulbecco’s Modified Eagle’s Medium, Lonza, Basel, Switzerland) culture medium for HaCaT, HepG2, HT29, MCF7, and A375, and RPMI-1640 for H460, supplemented with 10% FBS, Penicillin/Streptomycin 100× (Euroclone, Devon, UK), Glutamax 100× (Invitrogen, Carlsbad, CA, USA) and non-essential amino acids 100× (Invitrogen). Phosphate buffer (PBS, phosphate-buffered saline, Ca^2+^ and Mg^2+^ free) and trypsin (Ca^2+^ and Mg^2+^ free) were supplied by Euroclone. The cells were plated 15 × 10^3^ perwell in 96-well tissue culture plates and allowed to attach for 24 h. Then, cells were treated with PECS dissolved in culture medium supplemented with 1% FBS at different concentrations for 48 h (50 μg/mL, 80 μg/mL, 110 μg/mL, 140 μg/mL, 170 μg/mL, and 200 μg/mL). PECS were dissolved in sterile H_2_O at 100 mg/mL.

### 4.7. Sulforhodamine B Assay

After 48 h of treatment with PECS, cell survival/proliferation was measured in the presence and absence of the extract in 96-well plates by a spectrophotometric dye incorporation assay using Sulforhodamine B (SRB). Cells were fixed with trichloroacetic acid (Sigma Aldrich) for 1 h and after staining for 30 min with 0.4% (*w*/*v*) SRB (Sigma Aldrich) dissolved in 1% acetic acid. The number of viable cells was directly proportional to the protein bound-dye formation, which was then solubilized with 10 mM Tris base solution pH 10.5 and measured by fluorometric assay ELISA at 540 nm (Bio-Rad, Hercules, CA, USA; Microplate Reader). All experiments were performed in duplicate and were repeated for three times. The IC_50_ (the median inhibitory concentration defined as the drug concentration at which cell growth was inhibited by 50%) was assessed from the dose-response curves.

### 4.8. Apoptosis Detection

The cells (1 × 10^6^) were harvested and washed twice with ice-cold PBS. Subsequently, the cells were labeled with an Annexin V and Dead Cell Assay kit according to the manufacturer’s instructions (Merck Millipore, Darmstadt, Germany). This assay is based on the phosphatidylserine (PS) detection on the apoptotic cells surface, using fluorescently-labeled Annexin V in combination with the dead cell marker 7-aminoactinomycin D (7-AAD). The apoptotic ratio was calculated by identifying four populations: (i) viable cells, not undergoing detectable apoptosis: Annexin V (−) and dead cell marker (−); (ii) early apoptotic cells: Annexin V (+) and dead cell marker (−); (iii) late apoptotic cells: Annexin V (+) and dead cell marker (+); and (iv) cells that died through non-apoptotic pathway: Annexin V (−) and dead cell marker (+). The samples were counted by a Muse™ Cell Analyzer (Merck Millipore, Billerica, MA, USA) and analyzed by software provided by Merck Millipore.

### 4.9. Cell Cycle Assay

The Muse™ Cell Cycle Assay uses a premixed reagent. It contains the nuclear DNA intercalating stain propidium iodide (PI) and RNAse A in a proprietary formulation. PI discriminates cells at different stages of the cell cycle, based on differential DNA content in the presence of RNAse to increase the specificity of DNA staining. After the treatment with PECS, the cells were washed with PBS and, after centrifugation and removing of the supernatant, 1 mL of ice cold 70% ethanol was added to the re-suspending cell pellet. The samples were capped and frozen at −20 °C for at least 3 h prior to staining. Subsequently, the ethanol-fixed cells were subjected to several washes in PBS. Finally the pellet was suspended and incubated with 200 μL of Muse™ Cell Cycle Reagent for 30 min at room temperature, in the dark. After staining, the cells were processed for cell cycle analysis.

### 4.10. Mitochondrial Membrane Depolarization

Measurement of changes in mitochondria membrane potential (∆ψm) was performed with the Muse MitoPotential Assay kit™ (EMD Millipore Bioscience, Billerica, MA, USA). The assay utilizes the MitoPotential dye, a cationic, lipophilic dye to detect changes in the mitochondrial membrane potential, and 7-AAD as an indicator of cell death. High membrane potential drives the accumulation of MitoPotential dye within the inner membrane of intact mitochondria resulting in high fluorescence, while cells with depolarized mitochondria demonstrate a decrease in fluorescence. After the treatment, the cells were incubated with the fluorescent dyes and the percentage of depolarized cells (depolarized live + depolarized dead) was determined by a Muse Cell Analyzer. Muse MitoPotential Assay measures two important cell health parameters’ changes in mitochondrial potential, considered an early hallmark of apoptosis and cellular stress, namely cellular plasma membrane permeabilization or cell death. Briefly, cells, after the treatment with PECS, were harvested and the cell pellet was suspended in the assay buffer. MitoPotential dye working solution was added and the cell suspension incubated at 37 °C for 20 min. After the addition of Muse MitoPotential 7-AAD dye and incubation for 5 min, changes in DYm and in the cellular plasma membrane permeabilization were assessed using the fluorescence intensities of both the dyes analyzed by the Muse Cell Analyzer and flow cytometry. 

### 4.11. Bio-Plex Assay

Cytokines, chemokines, and growth factors in cellular supernatants after treatment with PECS were evaluated by the multiplex biometric ELISA-based immunoassay, containing dyed microspheres conjugated with a monoclonal antibody highly specific for a target protein. In particular, the following cytokines present in the Human Cytokine 27-Plex panel (Bio-Plex Bio-Rad) were evaluated: IL-1β, IL-1ra, IL-2, IL-4, IL-5, IL-6, IL-7, CCL2, CCL11, CXCL10, CXCL8, IFN-γ, IL-9, IL-10, IL-12 (p70), IL-13, IL-15, IL-17, basic FGF, G-CSF, GM-CSF, MIP-1α, MIP-1β, PDGF-ββ, RANTES, TNF-α, and VEGF. Protein levels were determined using a Bio-Plex array reader (Luminex, Austin, TX, USA) that quantifies multiplex immunoassays in a 96-well format with very small fluid volumes. The level of analytes was calculated using a standard curve, with the software provided by the manufacturer (Bio-Plex Manager Software, version 5.1). The cytokines expression levels, obtained as fluorescence intensities (FI), and evaluated in treated and untreated supernatants, were compared by *t*-test and fold change (obtained as ratio between FI (treated)/FI (untreated)). In particular, the values of *p* < 0.05 and fold change <0.5 and >1.5 were considered to be statistically significant.

### 4.12. ^1^H-NMR Metabolomic Analysis

HepG2 cells (~3 × 10^6^ cells/well) were seeded into 96-well cell culture plates and cultured overnight. Based on the aforementioned cytotoxicity, apoptosis, mitochondria depolarization, and cell cycle data, the cells was treated with PECS at the IC_50_ concentration. At 48 h cells underwent trypsin digestion and were then twice rinsed with PBS-D_2_O. These cell pellets were refrigerated at −80 °C. The cell pellets were re-suspended in 170 μL of H_2_O and 700 μL of methanol. The cells were sonicated for a period of 30 s. Then, 350 μL of chloroform was added and samples were mixed on an orbital shaker in ice for 10 min. H_2_O/chloroform (350 μL, 1:1, *v*/*v*) was added to each cell suspension and centrifuged at 10,000× g for 10 min at 4 °C. Thereafter, the aqueous phases were evaporated, and then dissolved in PBS-D_2_O with the pH adjusted to 7.20. The sodium salt of 3-(trimethylsilyl)-1-propanesulfonic acid (1% in D_2_O) was used as the internal standard. A 600-MHz BrukerAvance DRX spectrometer with a TSI probe was used. ^1^H-NMR spectra were collected at 300 K with the excitation sculpting pulse sequence to suppress the water resonance. A double-pulsed field gradient echo was used, with a soft square pulse of 4 ms at the water resonance frequency and the gradient pulses of 1 ms each in duration, adding 128 transients of 64 k complex points, with an acquisition time of 4 s/transient. Time domain data were all zero-filled to 256 k complex points and an exponential amplification of 0.6 Hz prior to Fourier transformation was applied.

### 4.13. Statistical and Pathway Analysis

The spectral 0.50–8.60 ppm region of ^1^H-NMR spectra was integrated in buckets of 0.04 ppm by the AMIX package (Bruker, Biospin GmbH, Rheinstetten, Germany). In detail, the water resonance region (4.5–5.2 ppm) was excluded during the analysis and normalized the bucketed region to the total spectrum area. To compare the spectra obtained for the polar phase of HepG2 cells before and after treatment, the fold change (FC) analysis and t-test were applied by the MetaboAnalyst tool [55]. The differences between the peak intensities were considered statistically significant using a FC threshold equal to 2 and p-value lower than 0.05. MetaboAnalyst 3.0 was used to identify the metabolic pathways more likely to be linked with the metabolic alterations induced under the three treatment comparisons [56]. The differential metabolites from each comparison were uploaded to the Pathway Analysis functionality on the MetaboAnalyst website (http://www.metaboanalyst.ca/). The *Homo sapiens* pathway library was used. The reference metabolome included all compounds in the selected pathways. The following pathway analysis algorithms were selected: Fisher test for over-representation analysis and relative-betweenness centrality for pathway topology analysis.

## Figures and Tables

**Table 1:** Apoptosis studies. The percentages of live, total apoptotic, and dead cells are reported for HepG2 cells before and after treatment with 137 μg/mL of PECS at 48 h.

HepG2 Cells	Live Cells	Early Apoptosis	Late Apoptosis	Dead
Untreated	93.80% ± 0.05%	2.33% ± 0.04%	2.48% ± 0.08%	1.39% ± 0.05%
Treated	34.51% ± 0.04%	1.30% ± 0.06%	50.91% ± 0.05%	13.28% ± 0.06%

**Table 2:** Cell cycle evaluation. The cell percentages in G0/G1, S, and G2/M phases are reported for HepG2 cells before and after treatment with 137 μg/mL of PECS at 48 h.

HepG2 Cells	G0/G1	S	G2/M
Untreated	46.6% ± 0.9%	8.8% ± 1.0%	44.6% ± 1.2%
Treated	61.8% ± 1.2%	3.9% ± 0.7%	33.0% ± 1.0%

**Table 3:** Mitochondria membrane potential cells after treatment with 137 μg/mL of PECS at 48 h.

HepG2 Cells	Live Cells	Depolarized Cells	Dead
Untreated	97.6% ± 0.8%	0.5% ± 0.07%	1.8% ± 0.05%
Treated	24.3% ± 0.9%	53.3% ± 1.0%	22.4% ± 0.8%

**Table 4:** Comparison between the fluorescence intensities (FI) evaluated in treated and untreated cells for the 27 cytokines in terms of fold changes and *p*-values.

Cytokines	Fold Change	*p*-Value
PDGF-ββ	0.92	0.18
IL-1β	0.96	0.20
IL-1ra	0.80	0.091
IL-2	1.27	0.26
IL-4	1.03	0.44
IL-5	0.86	0.15
IL-6	1.13	0.23
IL-7	0.89	0.11
IL-8	0.89	0.13
IL-9	0.94	0.26
IL-10	1.10	0.34
IL-12	1.07	0.48
IL-13	1.05	0.45
IL-15	1.15	0.19
IL-17	1.11	0.17
Eotaxin	0.81	0.090
FGF basic	1.47	0.088
G-CSF	1.00	0.52
GM-CSF	1.20	0.25
IFN-γ	0.93	0.29
IP-10	0.96	0.31
MCP-1	1.03	0.51
MIP-1α	0.84	0.088
MIP-1β	1.09	0.48
RANTES	1.19	0.37
TNF-α	0.46	0.047
VEGF	0.32	0.019

**Table 5:** List of ^1^H chemical shift (ppm) of metabolites found in HepG2 cells.

Metabolites	Group	Chemical Shift	Metabolites	Group	Chemical Shift
Leucine	δCH_3_	0.96	Phosphocholine	NCH_2_	3.6
Valine	γCH_3_	0.97	Valine	αCH	3.63
Valine	βCH_3_	1.04	Glycogen	C_2_H	3.65
Threonine	γCH_3_	1.20	Glycero-phosphocholine	NCH_2_	3.68
Isoleucine	γCH_2_u	1.24	Glycerol	C_1_H	3.68
Threonine	γCH_3_	1.32	α-Glucose	C_3_H	3.72
Lactate	βCH_3_	1.34	Alanine	αCH	3.75
Isoleucine	γCH_2_u	1.46	Glutamine	αCH	3.76
Alanine	βCH_3_	1.48	Glutathione	αCH	3.76
Leucine	βCH_2_	1.72	Glutamate	αCH	3.77
Lysine	δCH_2_	1.72	α-Glucose	C_6_H	3.78
Lysine	βCH_2_	1.90	Glycerol	C_2_H	3.82
Acetate	CH_3_	1.91	α-Glucose	C_5_H	3.84
Arginine	βCH_2_	1.91	Glycogen	C_6_H	3.86
Glutamate	βCH	2.06	Glycogen	C_5_H	3.88
Glutathione	βCH_2_	2.14	β-Glucose	C_6_H	3.90
Glutamine	βCH_2_	2.15	Creatine	CH_2_	3.92
Valine	βCH	2.28	Glycogen	C_3_H	3.92
Glutamate	γCH_2_	2.35	Phosphorylethanolamine	CH_2_	4.00
Malate	αCH	2.36	Phenylalanine	αCH	4.02
Glutamine	γCH_2_	2.43	Choline	αCH_2_	4.07
Glutathione	γCH_2_	2.62	Lactate	αCH	4.11
Aspartate	βCH_2_	2.66	Glycerol	1-CH_2_	4.11
Aspartate	β′CH_2_	2.79	Phosphocholine	OCH_2_	4.16
Glutathione	β″CH_2_	2.94	Threonine	βCH	4.26
Lysine	εCH_2_	3.03	Glycero-phosphocholine	OCH_2_	4.32
Creatine	NCH_3_	3.04	β-Glucose	C1H	4.64
Phosphorylethanolamine	CH_2_	3.2	β-Glucose	C1H	5.2
Phosphocholine	N(CH_3_)_3_	3.18	α-Glucose	C1H	5.24
Choline	N(CH_3_)_3_	3.19	α-Glucose	C1H	5.4
Arginine	δCH_2_	3.22	Uracil	CH	5.9
Glycero-phosphocholine	N(CH_3_)_3_	3.22	Fumarate	CH	6.52
β-Glucose	C_2_H	3.26	Histidine	C_4_H	6.91
Myo-Inositol	CH	3.28	Histidine	C_4_H′	6.99
β-Glucose	C_4_H	3.40	Tyrosine	C_2_,_6_H	7.15–7.2
α-Glucose	C_4_H	3.42	Tyrosine	C_2_,_6_H′	7.18
β-Glucose	C_5_H	3.47	Phenylalanine	C_4_H	7.33
β-Glucose	C_3_H	3.48	Phenylalanine	C_2_,_6_H	7.39
Myo-Inositol	CH	3.53	Phenylalanine	C_3_,_5_H	7.43
α-Glucose	C_2_H	3.54	Uracil	CH	7.53
Glycine	CH_2_	3.56	Histidine	C_2_H	7.78
Glycerol	1,3CH_2_OH	3.57	Formate	HCOO^−^	8.46
